# Comparative analysis of breast and lung cancer survival rates and clinical trial enrollments among rural and urban patients in Georgia

**DOI:** 10.32604/or.2024.050266

**Published:** 2024-08-23

**Authors:** TATIANA KURILO, REBECCA D. PENTZ

**Affiliations:** Winship Cancer Institute, Emory University School of Medicine, Emory University, Atlanta, 30322, USA

**Keywords:** Cancer, Cancer survival rates, Clinical trial enrollment, Rural patients, Health disparities, Barriers to clinical trial participation, Geographic disparities

## Abstract

**Objectives:**

Rural patients have poor cancer outcomes and clinical trial (CT) enrollment compared to urban patients due to attitudinal, awareness, and healthcare access differential. Knowledge of cancer survival disparities and CT enrollment is important for designing interventions and innovative approaches to address the stated barriers. The study explores the potential disparities in cancer survival rates and clinical trial enrollments in rural and urban breast and lung cancer patients. Our hypotheses are that for both cancer types, urban cancer patients will have longer 5-year survival rates and higher enrollment rates in clinical trials than those in rural counties.

**Methods:**

We compared breast and lung cancer patients’ survival rates and enrollment ratios in clinical trials between rural (RUCC 4-9) and urban counties in Georgia at a Comprehensive Cancer Center (CCC). To assess these differences, we carried out a series of independent samples *t*-tests and Chi-Square tests.

**Results:**

The outcomes indicate comparable 5-year survival rates across rural and urban counties for breast and lung cancer patients, failing to substantiate our hypothesis. While clinical trial enrollment rates demonstrated a significant difference between breast and lung cancer patients at CCC, no significant variation was observed based on rural or urban classification.

**Conclusion:**

These findings underscore the need for further research into the representation of rural patients with diverse cancer types at CCC and other cancer centers. Further, the findings have considerable implications for the initiation of positive social change to improve CT participation and reduce cancer survival disparities.

## Introduction

The rural populations have poor cancer survival outcomes compared to their urban counterparts [1]. The survival disparities have been attributed to the longer cancer pathway for rural populations due to attitudinal and awareness issues as well as the inherent inequalities in access to health and transport infrastructure between the rural and urban regions [2,3]. Further, rural-residing cancer patients have substantially lower CT participation rates due to several barriers, including poor transport system, limited health literacy and awareness, and longer travel time [4]. Research and knowledge on the extent of cancer survival disparities and CT enrollment differential are important as they offer useful insight to initiate relevant interventions to reduce inequalities in health access.

Medical science has experienced remarkable strides in cancer management, with clinical trials being pivotal for advancing efficacious therapies. However, patient engagement in clinical trials remains a challenge, with only 3% of adult cancer patients enrolling in such trials [5]. Addressing some of the barriers impeding patient participation is of paramount significance [6]. There is a paucity of studies focusing on clinical trial participation among rural cancer patients who grapple with inferior outcomes due to their restricted access to medical care, diagnostic delays, and a prevalence of emergency-based diagnoses [7]. The present study addresses this gap by assessing whether there is a difference in cancer survival rates among urban *vs*. rural Georgian patients with lung and breast cancer and whether there are different rates of participation in clinical trials conducted at a Comprehensive Cancer Center (CCC). The study also aims to assess whether enrolling in a CCC clinical trial impacts survival.

Our study’s three hypotheses are specified as:

*H*_A_1: Urban cancer patients will have a longer 5-year survival rate than rural cancer patients.

*H*_A_2: Urban cancer patients will participate in CCC clinical trials more frequently than rural patients.

*H*_A_3: Clinical trials participants will have longer survival rates than non-participants.

## Materials and Methods

### Research design and rationale

The study was conducted in Georgia, encompassing urban and rural locations [8]. The ages of the population studied were limited to 45–65 years because this study focused on the middle-aged cancer population, given the rising incidence rates of cancer in this age group [9,10]. Participants of all sexes, including male, female, and non-binary individuals, were included in this study. There was no randomization in this research. Power analysis was performed to determine the ideal sample size. The sample consisted of one-thousand, three-hundred and sixty-seven lung cancer patients (n = 1367) and two-thousand, two-hundred and sixty-four breast cancer patients (n = 2264). The sample was randomly selected in terms of rural-urban residence. For lung cancer patients, 1147 participants were urban patients while 221 were rural patients. Among the breast cancer patients, 2258 were from urban areas while only six were from rural areas.

### Hypothesis 1 methods

Data to determine the 5-year survival rates for each rural-urban classification scheme (RUCC) level was taken from Georgia Cancer Registries for lung and breast cancer from 2010 to 2016. RUCC has nine categories, with 1–3 representing metropolitan areas of differing population sizes and 4–9 representing nonmetropolitan regions characterized by population size and proximity to metropolitan areas [11].

We compared the average 5-year survival rates for breast and lung cancer patients in rural and urban Georgia counties using independent sample *t*-tests. Before conducting the *t*-test analysis, we ensured the data met certain assumptions. First, the homogeneity of variance using Levene’s test was assessed, which returned non-significant results for both breast cancer (F = 4.91, *p* = 0.06) and lung cancer (F = 2.44, *p* = 0.16) tests. These findings validate that the assumption of variance homogeneity was met [12].

Additionally, we examined the normality of the data distribution using Shapiro-Wilk’s normality tests. The tests indicated non-significance for both breast cancer (W = 0.88, *p* = 0.16) and lung cancer (W = 0.92, *p* = 0.42) tests, suggesting that the survival rates approximated a normal distribution. With these assumptions confirmed, we proceeded to conduct independent sample *t*-tests.

### Hypothesis 2 methods

We compared the frequency of rural patients in clinical trials to urban patients participating in clinical trials at the CCC in Georgia. The comparison was done between Georgia rural and urban patients with lung and breast cancer enrolled in CCC clinical trials, and Georgia rural patients with lung and breast cancer who were patients at the CCC but not enrolled in CCC clinical trials through a quantitative, non-experimental comparative application.

A series of Chi-Square tests of independence were conducted. The proportion of the population engaged in clinical trials was the dependent variable, while urban/rural designation and breast/lung cancer diagnosis constituted the independent factors.

### Hypothesis 3 methods

Five-year relative cancer-related data were collected from the SEER Tumor Registry, CCC Tumor Registry (CCC), and OnCore subject accrual data (CCC). The Surveillance, Epidemiology, and End Results (SEER) Program of the National Cancer Institute (NCI) is an authoritative source of information on cancer incidence and survival in the United States. SEER currently collects and publishes cancer incidence and survival data from population-based cancer registries covering approximately 35% of the U.S. population [13]. Since this registry contains data from 1975 to 2017, the information utilized was from 2010 to 2016 (6 years). The OnCore Enterprise Research system, or OnCore, is a vendor-supported Clinical Trials Management System (CTMS).

OnCore allows research teams to track subject details for specific trials. OnCore was used in the study to access data on rural and urban CCC patients with lung and breast cancer enrolled in clinical trials [14]. The procedures followed were in accordance with the ethical standards of the Helsinki Declaration. The approval of the Emory University Institutional Review Board (IRB) Committee was obtained before data collection and statistical analysis [15]. The IRB approval number is STUDY00004364. The IRB has granted a complete waiver of HIPAA authorization and informed consent. Protected Health Information of which use or access has been determined to be necessary by the IRB: data related to cancer type and survival outcomes from OnCore, Winship Tumor Registry, Georgia Cancer Registry, Rural Georgia SEER Registry (zip codes are only HIPAA identifier).

## Results

### Hypothesis 1 results

#### Rural *vs*. urban area Georgian patients’ cancer survival rates

**Breast Cancer:** Of the patients with breast cancer living in urban areas, 89.13% were surviving at the 5-year mark. In contrast, 85.40% of the patients with breast cancer living in rural areas were surviving at the 5-year mark (Table 1). The *t*-test analysis was conducted to determine if the observed survival rates in urban and rural areas were significantly different. The *t*-test outcomes (Table 2) indicated that the disparity in breast cancer survival rates was not statistically significant (*t*(7) = 2.23, *p* = 0.06).

**Table 1:** Descriptive statistics: 5-year breast and lung cancer survival rates in urban and rural counties

Variables	RUCC	Mean	Standard deviation	*p* value
5-year breast cancer survival rates (%)				
Urban	1–3	89.13	0.31	0.061
Rural	4–9	85.40	2.79	
5-year lung cancer survival rates (%)				
Urban	1–3	23.60	2.46	0.271
Rural	4–9	22.25	1.07	

**Table 2:** Independent sample *t*-test results for difference in breast and lung cancer survival rates in urban and rural counties

	Mean difference	*t*-statistic	Degrees of freedom	Probability value	95% confidence interval
Breast cancer survival rate (%)	3.73	2.234	7	0.061	–0.22 to 7.678
Lung cancer survival rate (%)	1.35	1.196	7	0.271	–1.32 to 4.02

**Lung Cancer:** Comparatively, lung cancer survival rates were notably lower than breast cancer in both rural and urban counties in Georgia. Of the lung cancer patients in urban counties, only 23.60% were surviving at 5 years while 22.25% of lung cancer patients in rural counties were surviving at the 5-year mark.

The estimated mean difference did not exhibit statistical significance at the 5% level (*t*(7) = 1.20, *p* = 0.27). The results meant that there was no evidence to validate the hypothesis that the lung cancer survival rates were significantly higher among urban patients compared to rural patients.

### Hypothesis 2 results

#### Rural *vs*. urban area Georgian cancer patients’ clinical trials enrollment

**Lung Cancer:** There were 1,367 lung cancer and 2,264 breast cancer patients who were treated at the CCC during the period of interest, 2010 to 2016. When comparing the clinical trial enrollment rate by region, 8.2% of lung cancer patients from urban areas were enrolled in clinical trials (CT) compared to 5.5% of patients from rural areas (Table 3). The chi-square results are summarized in Table 4. The test was not significant (χ(1) = 1.94, *p* = 0.16) indicating the absence of differential CT enrollment rate between urban and rural lung cancer patients.

**Table 3:** Frequency of patients enrolled in CT by cancer type and urbanization

Cancer/Clinical trial enrollment	Urban	Rural
Lung cancer		
Not enrolled	1053	208
Enrolled	94	12
Breast cancer		
Not enrolled	2071	1
Enrolled	187	5

**Table 4:** Chi-square tests of independence: CT participation and urbanization for lung and breast cancer

Cancer type	Chi-square	Probability value
Rural *vs*. urban CT for lung cancer	1.94	0.16
Rural *vs*. urban CT for breast cancer	43.43	<0.01
Lung *vs*. breast cancer CT rates	4.38	0.04

**Breast Cancer:** The analysis was repeated for breast cancer patients, among whom, 83.3% of rural patients were involved in clinical trials compared to 8% of urban patients. Even though chi-square analysis indicates a significantly different CT enrollment rate (χ(1) = 43.43, *p* < 0.01), the analysis was not reliable as one of the cells had an expected count of less than 5, violating a critical chi-square test assumption.

When considering the difference in the clinical trial enrollment rate of lung and breast cancer patients, breast cancer patients were shown to have a significantly higher CT enrollment rate compared to lung cancer patients (χ(1) = 4.38, *p* = 0.04). Overall, there was no evidence of significantly different CT enrollment rate of lung cancer patients in rural *vs*. urban areas while the sample of rural area breast cancer patients was too small to draw conclusions about the significance of the difference in CT participation rates. However, breast cancer patients participated in clinical trials at a higher rate than lung cancer patients.

### Hypothesis 3 results

#### Rural *vs*. urban area Georgian CCC CT participation cancer survival rates

A comparison of the 5-year survival rates among Georgia’s rural and urban patients enrolled in clinical trials, showed no significant difference based on *t*-test analysis (see Tables 5 and 6). Individual-level data were examined to assess this within a Comprehensive Cancer Center (CCC) in metropolitan Georgia. The results for the breast cancer patients indicate that on average, CCC clinical trial participants had higher cancer survival rates (M = 7.92, SD = 3.06) compared to non-participants (M = 7.46, SD = 3.89). The outcome of independent samples *t*-test confirmed that the difference in breast cancer survival rates by CCC clinical trial participation for both rural and urban patients was not statistically significant at 5% (*t*(249.58) = −1.93, *p* = 0.054).

**Table 5:** Descriptive statistics of 5-year breast and lung cancer survival by CCC participation

Variables	Number	Mean	Standard deviation
Breast cancer survival rates			
Participants	192	7.92	3.06
Non-participants	2072	7.46	3.89
Lung cancer survival rates			
Participants	106	3.71	2.96
Non-participants	1261	3.47	3.39

**Table 6:** Independent sample *t*-test results for difference in breast and lung cancer survival rates by CCC participation

Variable	*t*-statistic	Degrees of freedom	*p* value	95% confidence interval
Breast cancer survival rate	–1.93	249.58	0.054	–0.93 to 0.01
Lung cancer survival rate	−0.80	128.19	0.43	–0.84 to 0.36

Similarly, for the lung cancer patients, on average, the CCC clinical trial participants had higher cancer survival chances (M = 3.71, SD = 2.96) compared to non-participants (M = 3.47, SD = 3.39). The findings based on the independent samples *t*-test analysis indicated that there was no significant difference in lung cancer survival rates for rural and urban patients by CCC clinical trial participation (*t*(128.19) = −0.80, *p* = 0.43).

## Discussion

The analysis results did not support the hypotheses of higher breast and lung cancer survival rates for urban patients compared to rural patients. There was no evidence to support H_A_1 indicating that in Georgia, there are comparable cancer survival rates among urban and rural patients. However, these findings contrast the insight based on earlier evidence [16], which showed that cancer mortality is higher in rural compared to urban areas of the USA.

Further, there was a significant disparity in the clinical trial enrollment rate of breast cancer and lung cancer patients. For lung cancer, urban areas had a higher mean enrollment percentage than rural areas. However, this difference was not statistically significant. In contrast, for breast cancer, rural areas had a much higher mean enrollment percentage than urban areas. Even though the difference was statistically significant, the small sample size resulted in the violation of a key chi-square assumption making the finding unreliable. The results are inconsistent with the outcome of previous studies [17,18], which showed that breast cancer patients from rural areas had higher total intervals and longer diagnostic intervals compared to their urban counterparts. Health literacy and attitudinal factors were the most important factors that contributed to the lower CT enrollment rate among rural patients.

There was no substantial evidence to support H_A_2 based on the findings of the independent samples *t*-test. For breast and lung cancer patients in rural and urban Georgia, there were comparable cancer survival rates between CCC clinical trial participants and non-participants. The results indicate that participation in CCC clinical trial did not have a significant effect on breast and lung cancer survival outcomes. The results are inconsistent with the findings of previous studies [19] where it has been noted that participation in clinical trials offers between cancer survival chances due to access to state-of-the-art cancer care that exceeds the standard care guidelines.

The study aimed to close the literature gap by comparing the survival rates and the enrollment ratios of breast and lung cancer patients from rural counties to those from urban counties. The presented data and statistical analysis provide valuable insights into the differences in cancer survival rates and clinical trial enrollment between rural and urban areas in Georgia. While it is commonly assumed that cancer patients in urban areas fare better than cancer patients in rural areas, the lack of significant differences in the 5-year survival rates and the clinical trial enrollment rate of breast and lung cancer patients indicates that for this sample the stated assumption was not substantiated. The lack of significant differences in 5-year survival rates between rural and urban patients could be due to a variety of factors.

Within the CCC setting in Georgia, it is possible that the quality of care and access to treatment are relatively uniform across rural and urban patients, leveling the survival rates. The CCC has established three network sites throughout Georgia giving direct access to CCC specialty physicians to community physicians affiliated with the three sites. Two sites are in RUCC 3 counties and one in RUCC 4 county, whereas the CCC is in RUCC 1 county. Having dispersed network sites could help community physicians improve quality of care [20].

Several factors could contribute to higher breast cancer clinical trial enrollment. One possibility is the difference in the level of public awareness and support for different types of cancer. Breast cancer awareness has received significant attention and support due to the active advocacy of national breast cancer organizations, such as groups like the Susan G. Komen Foundation [21]. This has led to increased public awareness, fundraising, and early detection efforts for breast cancer. Lung cancer awareness, while increasing, faces challenges related to stigma and limited early detection options [22]. Future research should explore these potential factors in more detail to provide a comprehensive understanding of the observed results.

Additionally, within the CCC dataset, a distinctive disparity in patient distribution was noted. Among lung cancer patients, 19% originated from rural counties, while 81% were from metropolitan areas. For breast cancer patients, a mere 0.3% hailed from rural locales, with the overwhelming majority (99.7%) originating from metropolitan regions. The underrepresentation of rural patients with breast and lung cancer in the CCC compared to the state-level dataset highlights lack of geographic coverage, and differences in ability to access the CCC facility, which is located in a metropolitan area of Georgia. The biased sample composition could help explain the present research problem where breast and lung cancer patients from rural areas tend to have low CT enrollment rates and have longer diagnostic intervals.

This difference suggests rural individuals diagnosed with cancer may face unique challenges accessing Comprehensive Cancer Centers (CCCs), which are commonly located in urban areas. The necessity of traveling long distances to reach these urban centers may influence their healthcare decisions. Factors such as limited access to transportation, financial constraints, and the physical toll of travel may deter rural patients from seeking care at CCCs, even if these centers offer advanced treatments and clinical trials.

The insights gleaned from this research hold the potential to inform decision-making within the CCCs, guide patient education initiatives, facilitate networking and outreach efforts, secure grants, and drive future studies and developments. With a bias on lung and breast cancer, the results and findings depict the prevalence and survival rates in urban and rural areas. Future studies could delve into the specific challenges rural patients face when seeking care at urban CCCs, quantify the impact of travel distance on healthcare decisions, and evaluate the effectiveness of interventions aimed at mitigating this barrier.

## Limitations of the Study

This study encountered limitations such as the lack of more recent Rural-Urban Continuum Codes (RUCC), though the available dataset remains pertinent as of its last update on December 10, 2020. Additionally, reliance on secondary data precluded comprehensive exploration of all factors impacting rural populations. The study’s temporal scope of five years might not capture longitudinal changes, and the focus on Georgian CCC trials restricts generalizability to broader populations. Notably, this analysis omitted consideration of urban and rural Georgian populations’ racial and socioeconomic statuses. These factors are key confounding factors as they influence cancer survival outcomes [23,24]. However, given the secondary dataset limitation and the need to safeguard participants’ privacy, it was not possible to obtain detailed information on participants’ age, gender, racial profile, and socioeconomic statuses.

## Conclusion

The study findings provide insights into cancer survival rates and clinical trial enrollment trends among rural and urban patients in Georgia. Despite the widely believed assumption that rural patients receive subpar cancer care compared to their urban counterparts, the results of the present study indicated that urban patients did not have a superior survival rate and clinical trial participation rate than rural patients. While limitations were present, the study contributes to understanding the nuances of cancer care in different geographic settings.

The study findings also contribute to highlighting the extent of health inequity specifically with respect to breast and lung cancer care across the rural and urban population. Further, the findings have considerable implications in informing the need for a positive social change as a proactive intervention to improve cancer survival outcomes and CT participation/engagement for both rural and urban cancer patients. There is a need for future research encompassing diverse populations and comprehensive variables to enhance our understanding of the complex dynamics between cancer survival outcomes, CT participation, and urbanicity.

## Data Availability

The data underlying this article are available in NIH SEER Research Database SEER Incidence Data, 1975–2020 (cancer.gov). Additional data underlying this article were provided by Winship Cancer Institute of Emory University under permission. Data will be shared on request to the corresponding author with permission of Winship Cancer Institute.
